# Design of Spherical Nucleic Acids: From Fast Synthesis to Structural Engineering

**DOI:** 10.3390/biom16081105

**Published:** 2026-07-29

**Authors:** Yu Fan, Mei Tsz Jewel Chan, Jinyuan Liu

**Affiliations:** 1 Department of Biomedical Engineering, City University of Hong Kong, Kowloon, Hong Kong SAR 999077, China; yufan29@cityu.edu.hk (Y.F.); jewelchan2-c@my.cityu.edu.hk (M.T.J.C.); 2 Department of Polymeric Materials, School of Materials Science and Engineering, Tongji University, Shanghai 201804, China; 3 State Key Laboratory of Biopharmaceutical Preparation and Delivery, Institute of Process Engineering, Chinese Academy of Sciences, Beijing 100190, China

**Keywords:** spherical nucleic acids, aptamer, nanomedicine, gene therapy

## Abstract

Since the introduction of the spherical nucleic acid (SNA) paradigm in 1996, extensive research has been dedicated to this burgeoning field, yielding groundbreaking advances in biomedicine. Featuring a unique three-dimensional spherical nanoarchitecture composed of highly oriented, densely packed oligonucleotide layers conjugated to a solid or hollow core, SNAs possess extraordinary biological properties, including transfection-reagent-independent cellular internalization and enhanced resistance to nuclease degradation. These synthetic and foundational evolutionary milestones offer profound advantages for the rational design of targeted biomedical therapeutics. In this comprehensive review, recent breakthroughs in the synthetic methodologies and structural designs of SNAs, with a particular emphasis on gold-based templates, are systematically summarized and discussed. Furthermore, the core bottlenecks and future perspectives emerging at the intersection of artificial intelligence and high-throughput screening are highlighted to guide next-generation intelligent nanomedicine development.

## 1. Introduction

**Figure 1:**
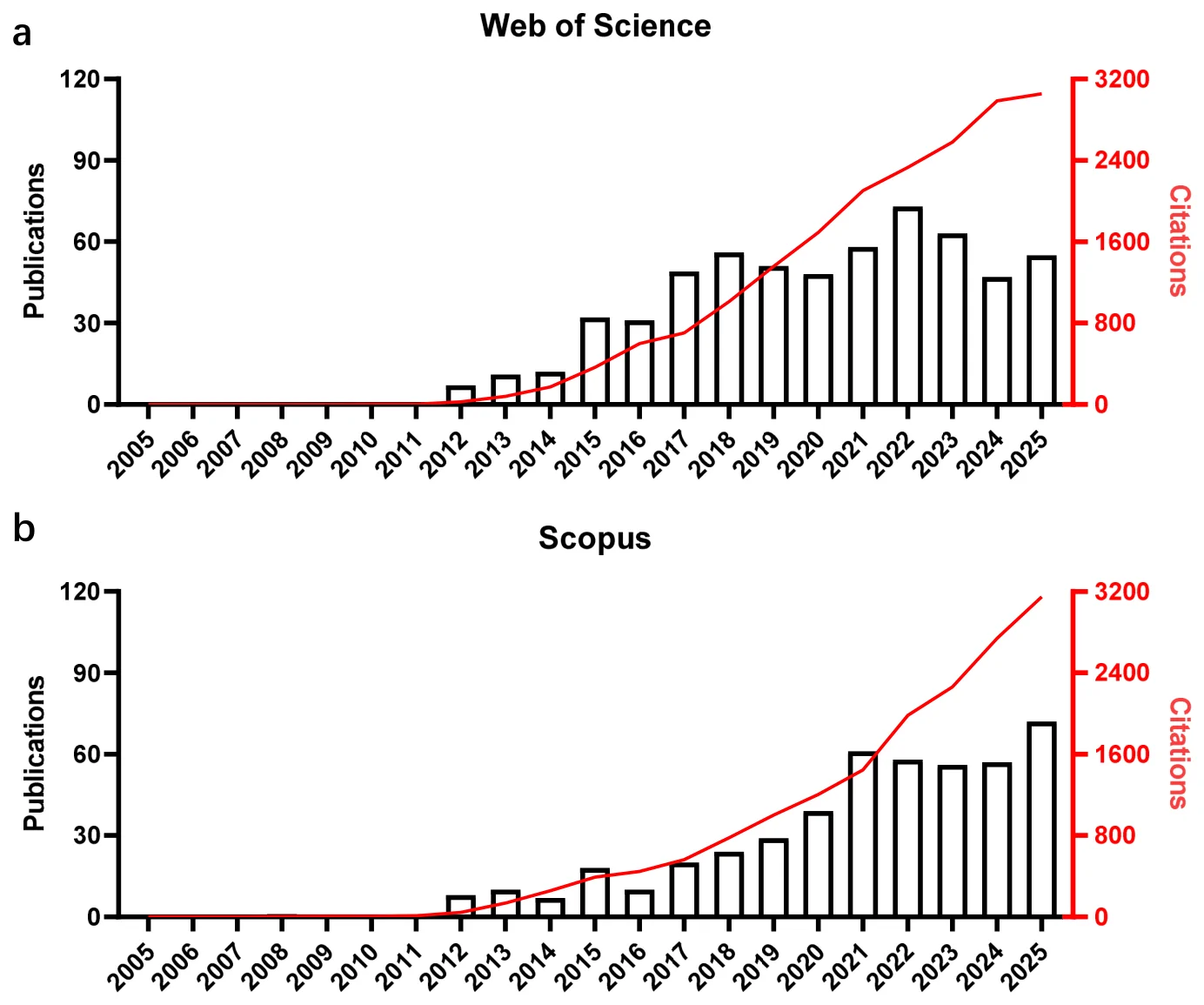
Publication record on SNAs and citation overview from 2005–2025. (**a**) A search of the Web of Science with “Spherical nucleic acids” (Topic) in all databases. (**b**) A search of Scopus with “Spherical nucleic acids” within the article title, abstract or keywords. The red curve shows the citation of articles.

Deoxyribonucleic acid (DNA) stands as one of the most fundamental biomolecules, capable of faithfully encoding genetic information with exceptional fidelity. The programmable sequence information intrinsic to DNA scaffolds guides the precise self-assembly of complex architectures and ensures the orchestrated regulation of targeted biological pathways, thereby enabling pre-engineered applications across diverse biological microenvironments. Over the past few decades, DNA origami [1,2], cages [3], and bricks [4] have emerged as prominent research hotspots, driving numerous recent synthetic breakthroughs. Beyond these established structural archetypes, a distinct modality of DNA nanotechnology utilizing spherical nucleic acids (SNAs) has garnered widespread attention, serving as the central focus of this review. Crucially, the SNA paradigm was pioneered by Mirkin and co-workers in 1996 [5]. The prototypical SNA architecture consists of a gold nanoparticle (AuNP) core surrounded by a densely packed, highly oriented shell of thiol-modified oligonucleotides covalently anchored to the metallic surface. Since this milestone, rapid synthetic evolution has yielded dozens of structurally distinct SNAs featuring highly diverse core and shell components [6,7]. Specifically, the core can be derived from various inorganic or organic matrices, while the surrounding oligonucleotide shell can be tailored with functional sequences to execute targeted biological roles. According to a search in Web of Science (Figure 1a) and Scopus (Figure 1b), publication trends for SNA-based biomedical materials have risen over the past twenty years, demonstrating that there remains vast territory for scientific exploration.

SNAs exhibit several unique physicochemical and biological characteristics that distinguish them from conventional nucleic acid nanostructures and confer clear therapeutic advantages across many application scenarios [8,9]. Furthermore, the polyvalent arrangement of the DNA shell facilitates highly cooperative molecular recognition, displaying a binding affinity for complementary targets significantly higher than that of their linear counterparts, thereby enabling precise interactions with target molecules. This highly ordered three-dimensional architecture allows for specific recognition by class A scavenger receptors, leading to rapid intracellular internalization through caveolin-mediated endocytosis in a non-toxic manner across nearly all cell types [10,11]. Like other nanostructured materials, the architectural and chemical properties of SNAs are heavily dictated by their initial synthesis parameters. Therefore, the capacity to independently tune these parameters with a profound understanding of their structure-function relationships remains essential for designing next-generation SNA-based nanomedicines. Recent research efforts have increasingly focused on developing rational design strategies to accelerate the exploration of these intricate structure-activity relationships and overcome the translational limitations of traditional platforms [12]. To provide a comprehensive overview, this review will systematically summarize the synthetic methodologies, innovative structural designs, and future clinical perspectives of SNA-based nanomedicines, with a particular emphasis on how advanced structural engineering can unlock their full translational potential.

## 2. Fast Synthesis of SNAs

**Figure 2:**
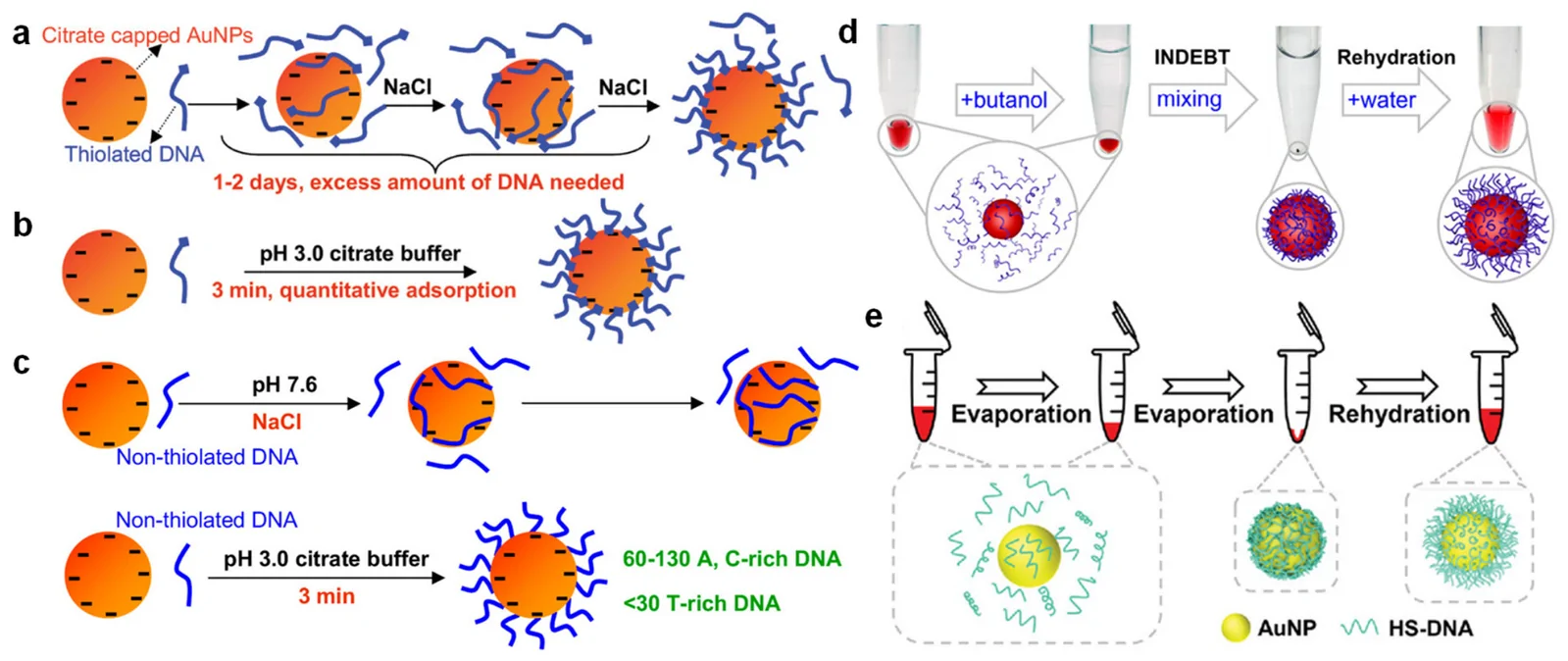
Schematics of attaching negatively charged thiolated DNA to negatively charged AuNPs using the salt aging (**a**) and the low pH-assisted (**b**) methods. Reproduced with permission [17]. Copyright 2012, American Chemical Society. (**c**) Schematic presentation of attaching nonthiolated DNA to AuNPs using the salt aging method (Upper) or at low pH (Lower). For nonthiolated DNA adsorption, it is generally accepted that DNA wraps around AuNPs, leading to a relatively low loading capacity. At low pH, a high capacity could be achieved, suggesting a different model of DNA adsorption. Adapted with permission [20]. Copyright 2012, American Chemical Society. (**d**) A typical INDEBT operation procedure. The sample was briefly centrifuged after mixing to view the dehydrated solid, which is not required normally. Reproduced with permission [21]. Copyright 2021, American Chemical Society. (**e**) Schematic illustration of an evaporative drying strategy for SNA synthesis. Reproduced with permission [24]. Copyright 2022, Wiley-VCH GmbH.

The primary bottleneck in fabricating SNAs stems from the severe electrostatic repulsions between the highly negatively charged DNA backbones and the nanoparticle surfaces. Prototypical 13 nm AuNPs, typically synthesized via citrate reduction, are electrostatically stabilized by the negative charges of weakly adsorbed citrate ions. Consequently, even low concentrations of electrolytes (e.g., 50 mM NaCl) can trigger irreversible AuNP aggregation. Because DNA molecules are highly polyanionic, this charge repulsion poses a formidable technical barrier; introducing high concentrations of NaCl to screen the surface charges often induces colloidal destabilization and AuNP aggregation before a dense layer of DNA can be successfully anchored. To circumvent this hurdle, Mirkin and co-workers pioneered the classic “salt-aging” protocol, wherein NaCl is step-wise added to the DNA/AuNP mixture [13]. Subsequent studies revealed that the packing density of the attached oligonucleotides directly correlates with the final NaCl concentration [14,15]. Crucially, the progressively anchored DNA layers enhance the steric stability of the AuNPs, allowing for the addition of higher salt concentrations to further maximize DNA loading. However, this iterative salt-aging process typically requires 12 days to yield a stable conjugate (Figure 2a). Furthermore, this methodology exhibits compromised efficiency when applied to larger AuNPs (e.g., 50 nm) and mandates tedious, multi-step salt additions [16]. To address the limitations of the time-consuming salt-aging method, several advanced, rapid synthetic strategies have been developed. For instance, a low-pH strategy elegantly leverages the accelerated grafting kinetics of DNA at increased acidity to outpace nanoparticle destabilization [17]. As illustrated in Figure 2b, this approach uniquely requires only a low-pH citrate buffer. More importantly, it facilitates the quantitative adsorption of multiple DNA sequences at predefined ratios, effectively eliminating the need for empirical payload quantification. Moreover, nonthiolated DNAs can be adsorbed onto AuNPs via the DNA bases. Adenine and cytosine have a strong affinity whereas thymine binds to the gold surface weakly [18]. At neutral pH, each 13 nm AuNP adsorbs just around twenty 12-mer DNAs even after overnight incubation [19]. This number is much lower than that achieved using thiolated DNA (up to 130 DNAs on each 13 nm AuNP [20]). For nonthiolated DNA adsorption (Figure 2c), salt aging at neutral pH typically yields low surface loadings [20], because the DNA strands tend to wrap around the AuNP through multiple bases, which limits the total number of attached strands. In contrast, the acidification method achieves an ultrahigh loading density within minutes by lowering the pH to 3. Under acidic conditions, DNA no longer lies flat but attaches primarily through one or a few terminal bases, standing upright on the surface.

Alternatively, Hao et al. developed an ultrafast synthesis based on instantaneous dehydration in butanol (INDEBT) [21], generating stable SNAs within seconds through a rapid, two-step solution-mixing workflow (Figure 2d). Briefly, an aqueous solution containing AuNPs and thiolated DNA is introduced into a butanol phase, where the volume of butanol is tuned to induce rapid, extraction-driven dehydration that drives interfacial assembly. Subsequently, a new aqueous phase is injected to rehydrate and harvest the self-assembled SNA solids. In a distinct approach, freezing-induced concentration has been utilized to facilitate DNA conjugation in the absolute absence of salts [22,23]. More recently, Ye et al. presented an evaporation-driven assembly strategy (Figure 2e), which achieves rapid and highly efficient packing of thiol-tagged DNA onto the NP surface at the critical moment of drying [24]. This preparation scheme is simple, robust, and easily scalable for bulk industrial manufacturing with minimal technical precautions. This evaporative drying process can operate across a wide temperature range, taking from several minutes to tens of minutes depending on the sample volume and heating rate.

A densely packed DNA shell may kinetically block the access of competing ligands and increase the overall bonding and colloidal stability of SNAs, even with nonthiolated DNA. Wang et al. reported a simple thermal evaporation process to dry AuNPs and nonthiolated DNA [25], which allowed rapid preparation of SNAs with DNA density and stability rivaling that with thiolated DNA. The preparation process involves simply heating the mixture of concentrated AuNPs (13 nm diameter) and DNA in a dry bath at 90 °C to fully evaporate the water in ~12 min. To reduce the time of heating, the AuNPs were first concentrated to ~10 μL by centrifugation before heating. Upon rehydration, the sample immediately returned to a red colloidal state. Since this method yielded stable and functional SNAs, Wang et al. explored the mechanism of conjugation [25]. A key to this method is the evaporation of water. To understand whether evaporation and heating are both required, they set up a control sample, where the tube was sealed during heating to minimize evaporation. Adding 1.0 M NaCl post-heating still triggers sample aggregation, offering clear evidence that thermal treatment alone fails to anchor DNA without an accompanying dehydration step. Even when the solution was concentrated from 20 μL down to 5 μL, salt-induced destabilization persisted despite the DNA concentration soaring to 200 μM. Failure of partial evaporation underscores complete dryness as a strict prerequisite for robust conjugate formation. At the molecular level, heating uncoils higher-order DNA conformations, forcing individual bases into direct competition for adsorption sites on AuNPs. Thermal energy selectively destabilizes weaker base-gold affinities, leaving only the highly resilient terminal adenine-gold linkages intact. Advantageously positioned over its internal counterparts due to minimal steric hindrance, terminal adenine drives the assembly toward maximum packing density as full desiccation forces the system into an ultra-concentrated state.

**Figure 3:**
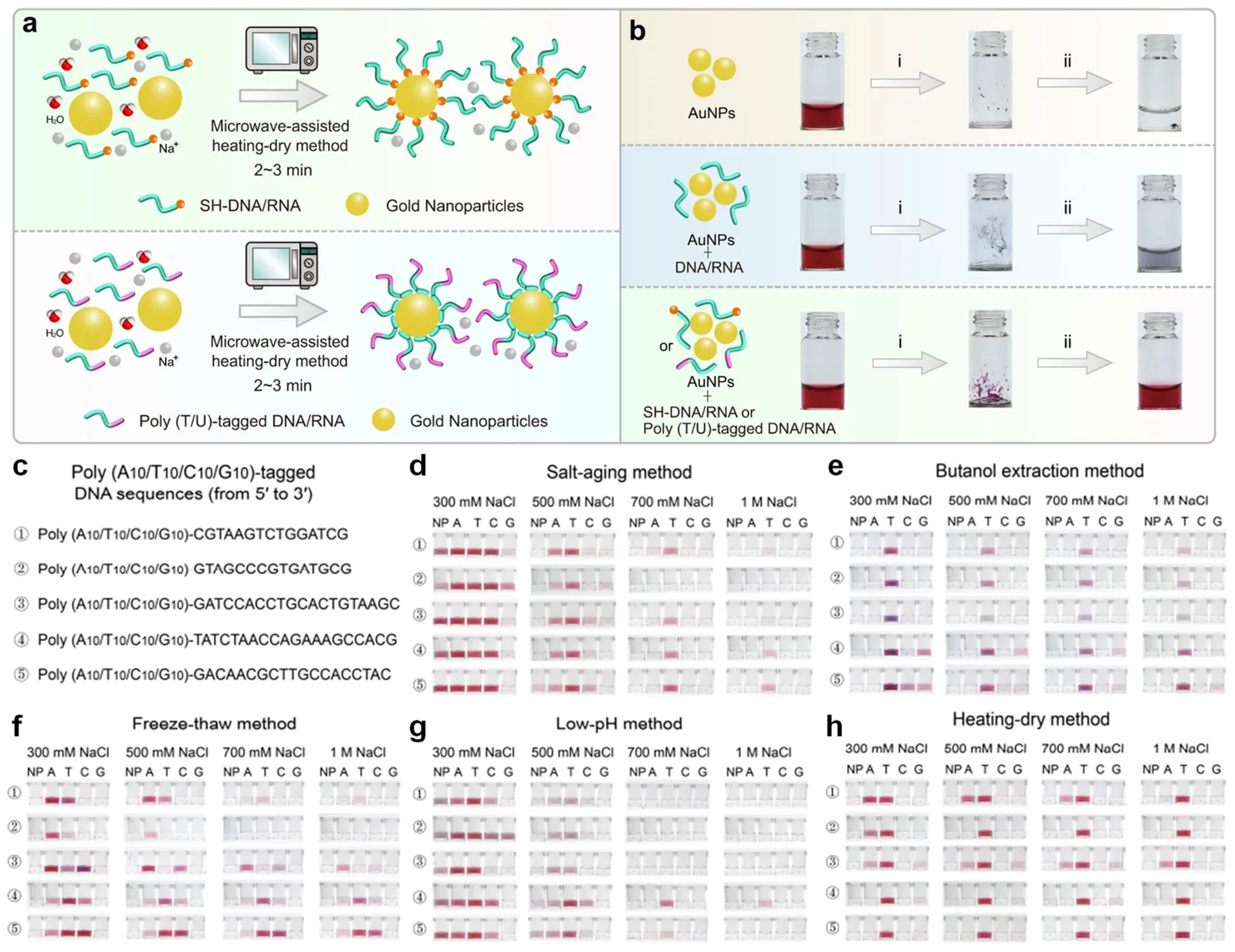
Fast construction of DNA/RNA-AuNP conjugates based on the MW-assisted heating-dry method. (**a**) Scheme of attaching thiolated and non-thiolated DNA/RNA to AuNPs using the MW-assisted heating-dry method, in which the heating-dry process is driven by a domestic microwave oven for 2–3 min. (**b**) Photographs of AuNPs before and after the heating-dry process. The bare AuNPs and random DNA/RNA sequence mixed AuNPs aggregated after heating-dry treatment. While poly (T/U)-tagged DNA-AuNPs retained monodispersity and seemed red after heating-drying and resuspension. (i) MW-assisted heating-dry; (ii) resuspending with water or buffer. Non-thiolated DNA-AuNP conjugation with various labeling methods. (**c**) Detailed sequences of six poly (A10/T10/C10/G10)-tagged DNA probes. Photographs showing the labeling results of the six poly (A10/T10/C10/G10)-tagged DNA probes using (**d**) the salt-aging method, (**e**) butanol extraction method, (**f**) freeze–thaw method, (**g**) low-pH method, and (**h**) MW-assisted heating-dry method. The DNA-AuNP conjugates were resuspended in 300 mM, 500 mM, 700 mM and 1 M NaCl solution, respectively. The MW-assisted heating-dry method exhibits maximum labeling efficiency and excellent sequence universality compared with other methods. Adapted with permission [26]. Copyright 2022, Springer Nature, Licensed under CC BY 4.0.

A recent strategy utilizes microwave (MW) radiation to achieve rapid, dehydration-driven nucleic acid conjugation onto AuNPs [26]. Because MW radiation is completely reflected by the metallic surface, it selectively heats and evaporates the surrounding aqueous solution. This heating-dry approach creates an ideal kinetic window for interfacial assembly by simultaneously unfolding the nucleic acid chains and concentrating the reaction volume. Consequently, thiolated oligonucleotides can be efficiently attached to AuNPs via a simple domestic MW protocol, yielding highly stable SNAs within minutes (Figure 3a). Although this evaporation regime requires poly(T/U) tags for preferential attachment—failing which random or untagged sequences lead to irreversible colloidal aggregation (Figure 3b)—it offers exceptional sequence generality when such tags are utilized. Documented across Figure 3c–h, the high efficiency, robust conjugate stability, and environmentally friendly nature of this method position it as a highly practical alternative for noble metal interfacial engineering.

## 3. Structural Engineering: Determinant for Various Biomedical Applications

The typical SNA structure is composed of four essential components from the inside out: a core, an attachment group, a spacer, and a recognition or functional sequence [27]. Because this spatial constitution decisively dictates the nanostructure’s function, selecting appropriate candidates for each component is crucial for tailored applications.

### 3.1. Evolutionary Trajectory of Core

**Figure 4:**
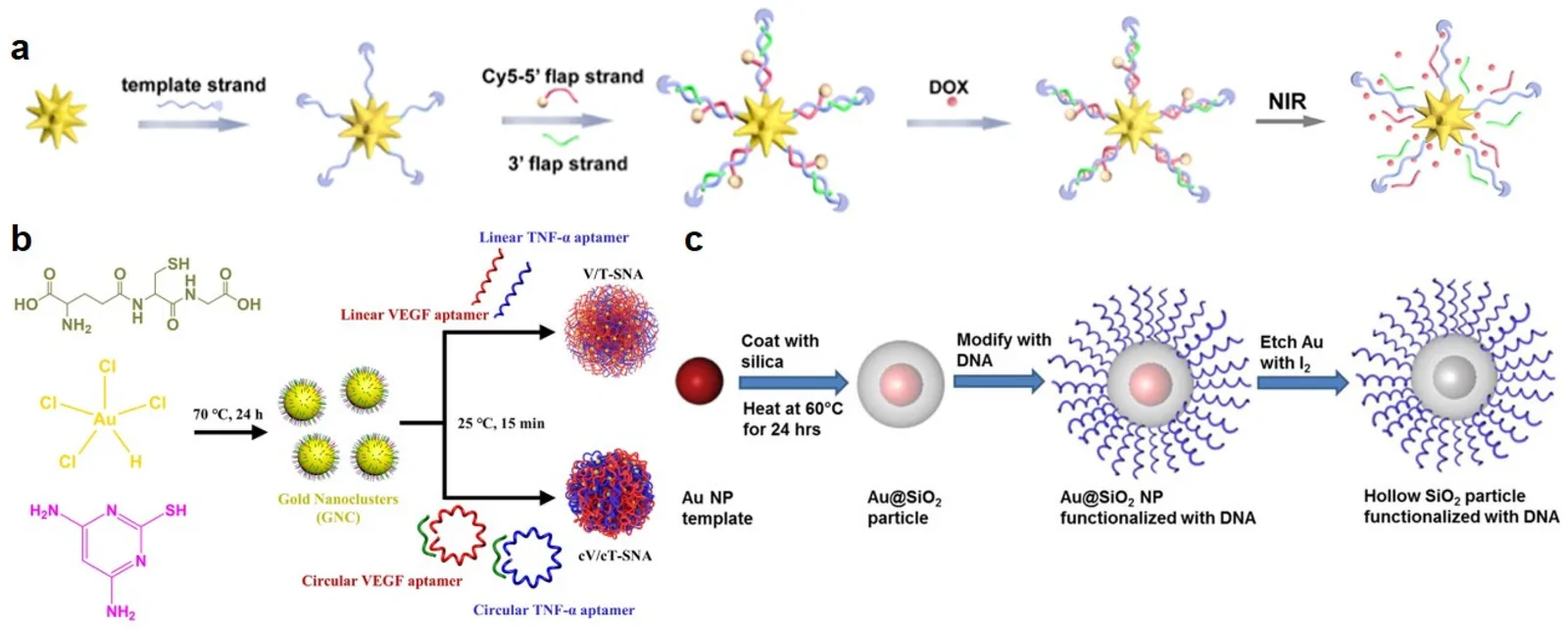
(**a**) Design of gold nanostar-conjugated DNAs as spherical nucleic acids for biomarker assay and controlled drug delivery and illustration of accelerated DOX release under NIR irradiation. Adapted with permission [32]. Copyright 2021, American Chemical Society. Schematic illustration of (**b**) the synthesis of cV/cT-SNA for psoriasis treatment by inhibiting excessive keratinocyte proliferation and reducing inflammatory responses. Adapted with permission [33]. Copyright 2025, American Chemical Society. (**c**) Synthesis of DNA-functionalized hollow SiO_2_ particles using gold nanoparticles as sacrificial templates. Reproduced with permission [34]. Copyright 2012, American Chemical Society.

Various synthetic strategies have been developed to functionalize these AuNP cores; for instance, oligonucleotide chains can be anchored onto AuNPs via covalent linkages (Au-S or Au-Se bonds [28,29]) or through the preferential adsorption of consecutive adenines (polyA) [30,31] onto the gold surface. To expand the functional scope of SNA cores, Li et al. introduced SNAs that were composed of gold nanostars (AuNSs) and oligonucleotide double strands [32]. Capitalizing on the high photothermal transduction efficiency of the AuNS core, this system generates localized heat upon light irradiation to dehybridize the DNA and trigger cargo release (Figure 4a). Moreover, Liu et al. addressed the challenge of skin stratum corneum penetration for psoriasis treatment with circular SNAs [33]. Their SNAs were assembled via electrostatic interactions between gold nanoclusters (~2.3 nm) and circularized oligonucleotides encoding vascular endothelial growth factor (VEGF) and tumor necrosis factor-alpha (TNF-α) aptamers (Figure 4b). During assembly, the multimeric circular aptamers bridged multiple gold nanoclusters into organized complexes. Compared with their linear counterparts, these circular SNAs exhibited markedly superior nuclease resistance, enhanced capture efficiencies, and deeper transdermal penetration. This robust penetration capability enabled the circular SNAs to effectively cross the stratum corneum to reach deep psoriatic lesions, where they specifically bound to VEGF and TNF-α to coordinate the inhibition of excessive inflammatory responses and keratinocyte proliferation. Alternatively, altering the core material entirely, Young et al. developed hollow silica-based SNAs [34]. This was achieved by coating AuNP templates with a thin silica shell (~15 nm) via the ammonia-catalyzed hydrolysis of tetraethyl orthosilicate, followed by oxidative dissolution of the gold core (Figure 4c). The resulting porous silica shell acts as a cross-linked scaffold for assembling oriented oligonucleotides, imparting these hollow SNAs with cooperative binding behavior and cellular uptake profiles comparable to their solid gold counterparts.

### 3.2. Architectural Modulation of the Nucleic Acid Shell

SNAs elegantly illustrate how engineered nucleic acid architectures can dictate critical biological processes, including receptor recognition and cellular trafficking. However, the inherent flexibility of the oligonucleotide shell often obscures the precise correlation between specific DNA structural features and their resultant biological responses. Crucially, the architecture of the nucleic acid shell functions not merely as a passive coating, but as an active, programmable determinant of biological fate.

**Figure 5:**
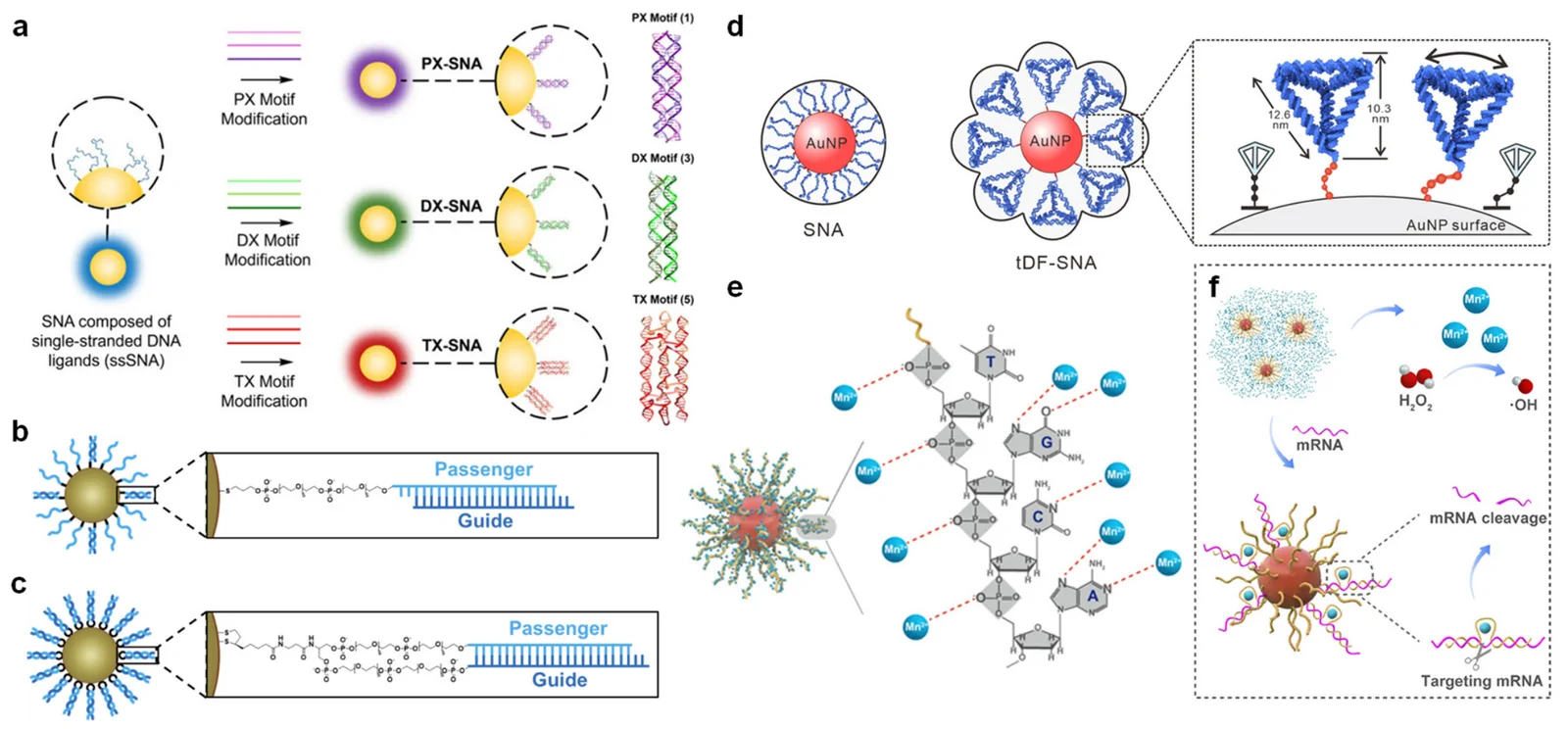
(**a**) SNAs composed of single-stranded DNA ligands (ssSNAs) can be modified with additional DNA nanostructure forming strands in the presence of divalent cations to form DNA nanostructures modified SNAs. Reproduced with permission [35]. Copyright 2026, American Chemical Society. Chemistry of siRNA-SNA Attachment Architectures. (**b**) Hybridized architecture, in which the passenger strand is attached to the core via a PEG linker with a thiol group. (**c**) Hairpin-like architecture, in which both strands are attached to the core via a PEG linker with a dithiol serinol group. Reproduced with permission [36]. Copyright 2022, American Chemical Society. (**d**) Schematic of the design for tDF-SNAs and SNAs. Adapted with permission [37]. Copyright 2024, Wiley-VCH GmbH. (**e**) Interaction of Mn^2+^ ions with multiple sites of DNAzyme on AuNPs. (**f**) A schematic illustration showcases the released DtMnP assisting in the generation of ROS and gene silencing. Reproduced with permission [38]. Copyright 2025, Wiley-VCH GmbH.

Ma et al. systematically investigated how well-defined DNA nanostructures assembled on preformed SNA surfaces influence cellular internalization [35]. Briefly, AuNPs were functionalized with double crossover (DX), paranemic crossover (PX), or triple crossover (TX) motifs to generate structurally modified SNAs (designated as DX-SNAs, PX-SNAs, and TX-SNAs, respectively). As illustrated in Figure 5a, each nanostructure was assembled via hybridization of four DNA strands, with an anchor strand initially conjugated to the gold surface to serve as a scaffold. These three architectures, varying in clustering density and spatial topology, were evaluated across diverse cell lines. Direct comparisons were made between SNAs possessing distinct clustering profiles (TX versus DX/PX) and those with identical clustering but divergent topologies (DX versus PX). All three multidimensional architectures exhibited significantly higher cellular uptake than conventional, single-stranded SNAs. Notably, TX-motif-modified SNAs demonstrated the highest cellular uptake, which directly correlated with their superior capacity for Ca^2+^ sequestration. Mechanistically, increased DNA clustering and higher crossover numbers elevate the local negative charge density, which enhances Ca^2+^ binding. The sequestered Ca^2+^ effectively neutralizes the phosphate backbone charges, reducing electrostatic repulsion with the cell membrane and facilitating interactions with Ca^2+^-binding proteins. Beyond enhancing internalization efficiency, these structured DNA motifs shifted the endocytic pathway from the caveolin-mediated route typical of traditional single-stranded SNAs to predominantly clathrin- and macropinocytosis-mediated pathways.

Building on the growing recognition that the nucleic acid shell dictates biological function, Mahajan and colleagues reengineered SNAs to act as direct agonists of innate immune sensors rather than conventional delivery vehicles for exogenous therapeutics [39]. Their platform comprises a 15 nm gold core densely functionalized with interferon-stimulatory DNA oligonucleotides. These nanostructures directly bind to cyclic GMP-AMP synthase, stimulating the catalytic production of endogenous cyclic dinucleotides and triggering the downstream Stimulator of Interferon Genes (STING) pathway. While conventional cyclic dinucleotide STING agonists suffer from poor cellular permeability and require invasive intratumoral injections, this dense SNA architecture exploits inherent cellular uptake pathways to efficiently deliver double-stranded DNA ligands intracellularly. Following internalization, a fraction of the DNA ligands escapes into the cytosol to induce sustained STING activation. Remarkably, these SNAs can be administered intranasally, leveraging the trigeminal nerve pathway to bypass the blood–brain barrier, accumulate in orthotopic glioblastoma tumors, and successfully reprogram the immunosuppressive tumor microenvironment. Consequently, the dense DNA shell functions as a standalone signaling entity capable of sensing and amplifying innate immune pathways without requiring secondary therapeutic payloads.

With an increase in stability and cellular uptake, SNAs have also shown immense promise as nanocarriers for small interfering ribonucleic acids (siRNAs), typically achieved by hybridizing siRNA duplexes onto the densely packed strands on the gold surface. Nevertheless, the therapeutic translation of siRNA-loaded SNAs remains bottlenecked by inefficient cytosolic delivery, primarily constrained by the inherently low stability of the hybridized duplexes. Prototypical hybridization designs frequently suffer from the premature dissociation of the guide strand from the passenger strand, prematurely depleting the payload of active siRNA duplexes before reaching target sites. Moreover, even after receptor-mediated endocytosis, these conventional siRNA-SNAs are routinely trapped within endo/lysosomal compartments rather than being released into the cytosol. To overcome this limitation, a novel SNA design was investigated [36], which directly attaches both siRNA strands to the SNA core through a single, continuous hairpin-shaped molecule to prevent guide strand shedding. In contrast to traditional hybridized architectures where only the passenger strand is directly anchored to the AuNP core and the complementary guide strand is loosely hybridized with low duplexing efficiency (Figure 5b), this unimolecular configuration incorporates a single RNA strand comprising the duplex payload and a loop region formed by poly(ethylene glycol) (PEG) spacers (Figure 5c). This structural modification achieves exceptionally high duplexing efficiency, effectively preventing premature guide strand dissociation and increasing the siRNA loading capacity onto the SNA core by four-fold compared with conventional designs.

Li et al. addressed the intertwined challenges of low siRNA duplexing efficiency and suboptimal endosomal escape by drawing inspiration from the structural topography of coronavirus spike proteins [37]. They engineered a 37-base pair triangular pyramidal tetrahedral DNA framework (tDF; ~10.3 nm in height and ~12.6 nm in length) that mimics the geometry and dimensions of the viral spike protein (Figure 5d). To facilitate core anchoring, one edge of the tDF was functionalized with a thiol-tagged single-stranded DNA spacer (ssDNA), which acts as a flexible stalk to secure the framework onto a 50 nm gold core via Au-S coordination. This biomimetic configuration replaces conventional flexible single-stranded DNA shells with highly rigid, densely packed tDF arrays. Linear oligonucleotides tend to lie flat against the nanoparticle surface, while this collapsed state severely penalizes subsequent hybridization entropically. In contrast, these spiky tDFs stand upright and undergo dynamic tilting. This architectural orientation minimizes steric hindrance and provides highly ordered, spatially accessible binding sites for siRNA capture, remarkably boosting duplex hybridization efficiency from ~20% to 95%. More importantly, the rigid, highly ordered framework actively modulates the cellular internalization pathway, shifting it from the conventional clathrin-mediated route (which typically targets payloads for lysosomal degradation) to a clathrin-independent engulfment pathway. This topological intervention directly facilitates efficient cytosolic release, which ultimately translates to an enhancement in therapeutic gene silencing. Concurrently, owing to these collective structural advantages, the intracellular delivery efficiency of siRNA molecules via these tDF-SNAs is improved by 1–2 orders of magnitude compared with traditional SNAs.

Xie et al. synthesized DNAzyme-functionalized AuNPs and subsequently employed a cryosynthesis method to grow a pH-responsive manganese carbonate shell around them [38]. In this design, the oligonucleotides served both as the therapeutic agent (the DNAzyme) and as a high-affinity scaffold for Mn^2+^ ions, leveraging the phosphate, nitrogen, and oxygen sites within the DNA backbone that exhibit strong coordination networks with Mn^2+^ (Figure 5e). The resulting nanoparticle functioned as a prodrug that remains inert at physiological pH but undergoes rapid dissolution within the acidic tumor microenvironment, simultaneously releasing the active DNAzyme and Mn^2+^ ions. As shown in Figure 5f, the liberated Mn^2+^ ions act as essential cofactors to activate the DNAzyme for specific mRNA cleavage, effectively addressing the persistent in vivo challenge of insufficient metal cofactors for DNAzyme catalysis. Furthermore, Mn^2+^ serves dual diagnostic and therapeutic roles as a magnetic resonance imaging contrast agent and a Fenton reaction catalyst, beautifully illustrating how SNA-directed mineralization can seamlessly integrate molecular imaging and targeted therapy for cancer theranostics.

Collectively, these diverse systems illustrate a clear progression in SNA architectural engineering, offering elegant design rules for the deliberate construction of future SNA-based nanomedicines. The highly programmable nature of DNA allows for the complete decoupling of historically conflicting design requirements. Consequently, researchers can independently optimize target binding, cellular internalization, or multi-cargo co-delivery by simply altering the nucleic acid’s connectivity and spatial presentation on the core surface. This exceptional structural flexibility positions SNAs not merely as a fixed class of materials, but as a versatile, programmable platform where the ultimate biological outcome is fundamentally written into the shape and sequence of the nucleic acid shell itself.

### 3.3. Next Generation of SNA

Although conventional SNA nanostructures feature the inherent stability and rigidity of nanoparticle templates, they do not preserve a precise structure at the molecular level; their particle size and colloidal stability are continuously influenced by the core, oligonucleotide composition, and preparation conditions. In retrospect, these molecularly undefined structures have limited the precise exploration of structure-property relationships. In response, a second generation of SNAs, molecular spherical nucleic acids (mSNAs) with well-defined chemical structures, has been explored. For example, Mirkin and colleagues reported a class of nanomolecular architectures composed of a molecular core (such as polyoctahedral silsesquioxane or C60) functionalized with exactly 8 or 12 pendant DNA strands [40]. These mSNAs achieved comparable nuclease resistance, cellular uptake, and gene regulation capabilities without requiring external antisense agents [41,42]. Nevertheless, mSNAs typically involve only a single DNA sequence, which inherently restricts their capacity to simultaneously execute active targeting and multi-gene regulation.

**Figure 6:**
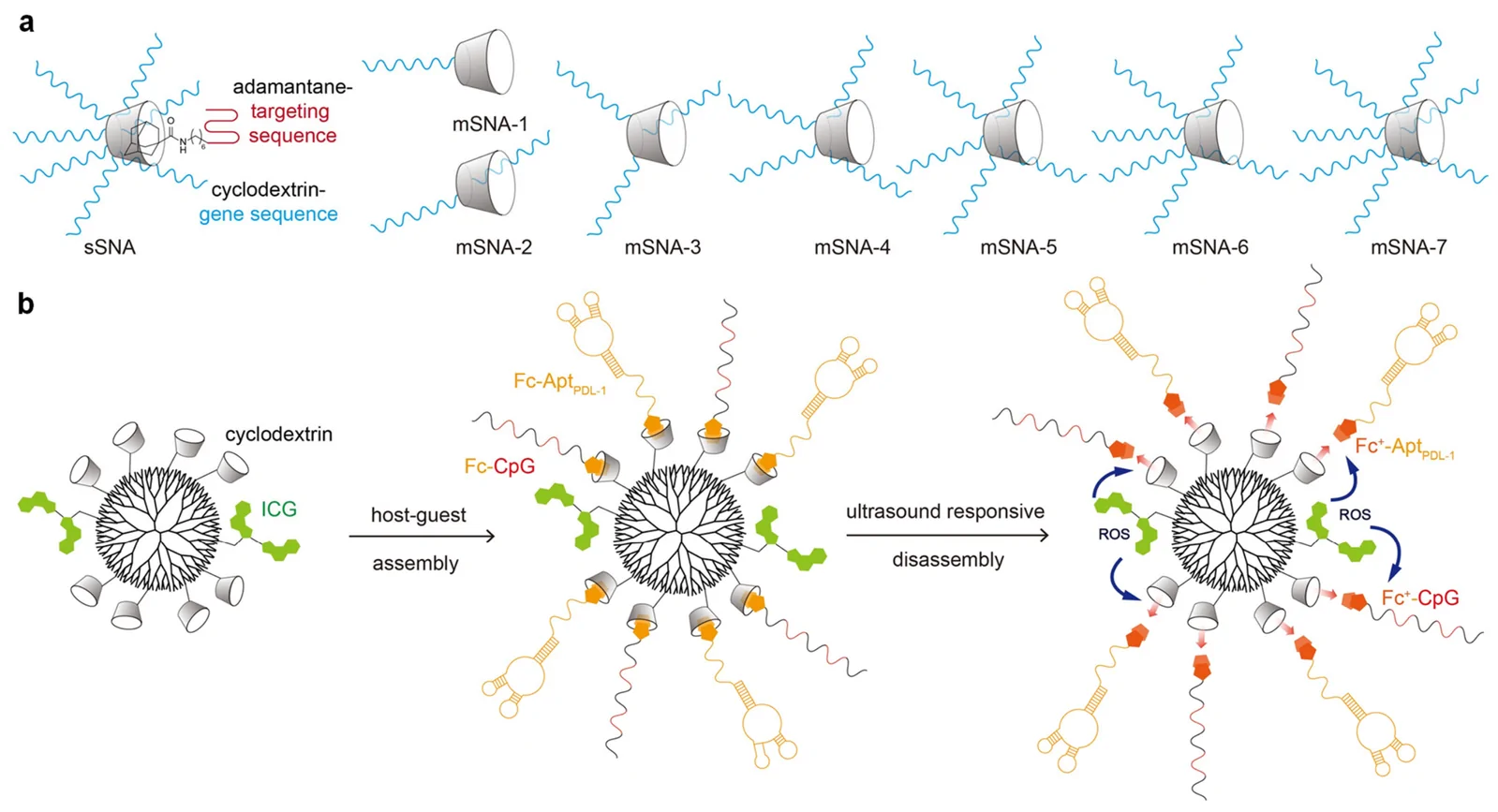
(**a**) The structural illustration of supra-SNAs and molecules-SNAs from mSNA-1 to mSNA-7. (**b**) Schematic illustration of host–guest supramolecular assembly and ultrasound irradiation-induced sSNA disassembly.

To expand this functional scope, Chen et al. developed a third generation designated as supramolecular spherical nucleic acids (sSNAs), which incorporate two distinct oligonucleotide sequences to achieve targeted delivery and gene regulation [43]. As shown in Figure 6a, the sSNA structures were constructed by coupling a targeting sequence-modified adamantane with human epidermal growth factor receptor 2 (HER2) antisense-functionalized β-cyclodextrin (β-CD). Therein, they employed click chemistry to synthesize precisely structured mSNAs with controlled valency, spanning from mono- to hepta-substitution (designated as mSNA-1 to mSNA-7 in Figure 6a). Consequently, the sSNAs composed of both anti-HER2 DNA strands and targeting sequences demonstrated significantly higher cellular uptake and superior anti-tumor efficacy compared to anti-HER2 sequences alone. Recently, Xu et al. proposed a host–guest supramolecular assembly strategy for the precise construction of sSNAs to treat oral leukoplakia (OLK) [44]. As illustrated in Figure 6b, β-CD and indocyanine green (ICG) were co-modified onto the termini of a poly(amidoamine) dendrimer possessing a well-defined spherical geometry. The resulting β-CD-grafted dendrimers enabled the radial arrangement of ferrocene (Fc)-tagged oligonucleotides through host–guest molecular recognition. Upon ultrasound irradiation, the sSNA generated cytotoxic reactive oxygen species to dissociate the supramolecular assembly, concurrently releasing CpG oligonucleotides and Programmed Death-Ligand 1 (PD-L1) aptamers. Thereafter, the liberated CpG enhanced antigen-presenting functions in dendritic cells and potentiated the activation of CD4+/CD8+ T cells. Simultaneously, the released PD-L1 aptamers bound to PD-L1 on OLK cells, blocking PD-1/PD-L1 checkpoint signaling to sustain T-lymphocyte activation. This orchestrated cascade ultimately inhibited pathological OLK cell hyperproliferation and established robust adaptive immune memory by expanding central and effector memory T-cell populations, thereby preventing OLK recurrence and its malignant progression to oral squamous cell carcinoma.

In short, well-defined molecular architectures provide a highly responsive and versatile SNA platform through host–guest supramolecular assembly strategies. The rapid evolution of SNAs is poised to redefine the frontiers of nanoscale materials chemistry. Specifically, molecular SNAs will enable atomically precise nanoarchitectures, while supramolecular SNAs will supply the essential dynamics and responsiveness required for next-generation smart biomaterials. Future SNAs will inevitably move beyond traditional gold nanoparticles to encompass diverse inorganic, organic, or hybrid cores. Concurrently, advances in oligonucleotide sequence design and surface grafting density will facilitate ultra-high loading of therapeutic oligonucleotides, gene-regulating motifs, and multimodal imaging probes. A central theme in the future of SNAs lies in the seamless integration of tailored molecular cores with programmable nucleic acid sequences via precise chemical bonds [45,46] or non-covalent physical interactions [47,48]. Ultimately, supramolecular assembly will empower the precise customization of vast SNA libraries, expanding the horizons of stimuli-responsive systems and DNA-mediated nanotheranostics.

## 4. Perspectives in Medicinal Application

We have systematically summarized the design principles and synthetic strategies of SNA-based biomedical materials, with a particular focus on the architectural modulation of their core templates and outer nucleic acid shells. Although significant research efforts have been devoted to the development of conventional SNAs, mSNAs, and sSNAs, achieving optimal design and fully realizing the biomedical potential of these nanostructures remain an evolving frontier that presents profound challenges.

### 4.1. Challenges and Limitations

#### 4.1.1. Nuclease Tolerance and Potential Toxicity

Although enzymatic catalysis is essential for functionalized SNAs to liberate free oligonucleotide strands and exert their therapeutic functions, premature enzymatic degradation must be strictly restricted. Prior to reaching target tissues or when distributed non-specifically within the body, SNAs must possess sufficient stability to minimize required dosages while maintaining functionality and mitigating off-target toxicity. The spherical architecture of SNAs, characterized by a densely packed oligonucleotide shell, naturally provides superior resistance to nuclease degradation, thereby overcoming a major bottleneck prevalent in conventional oligonucleotide-based therapies. In essence, while the SNA nanostructure remains a polyvalent DNA conjugate that cannot completely block nucleases from binding, its tightly packed spatial arrangement generates a high localized negative charge density. This dense negative charge induces severe steric hindrance and electrostatic repulsion that impedes the approach of nucleases, effectively suppressing their hydrolytic activity. Notably, when high-density SNAs are incubated with DNase I or DNase II for 24 h, the surface oligonucleotide density remains above 90% of its initial level [49]. This stability is strongly dependent on shell density; a decline in oligonucleotide packing on the SNA surface leads to a corresponding decrease in nuclease resistance. Seferos et al. systematically evaluated this correlation by incubating free DNA molecules and intact SNAs with DNase I to determine their respective enzymatic half-lives [50]. Their findings revealed that the half-life of SNAs was approximately 4.3-fold longer than that of free DNA. Subsequently, backfilling the surface with varying concentrations of PEG was utilized to artificially decrease the density of active oligonucleotide chains on the core. As the PEG concentration increased, the density of the oligonucleotide chains gradually decreased, which concurrently compromised the half-life of the nanostructures. Specifically, when the oligonucleotide density was reduced to approximately half of its original value, the enzymatic half-life similarly decreased by half, further demonstrating that high-density surface passivation is the deterministic factor governing the resistance of SNAs to nucleolytic degradation.

Additionally, a critical concern requiring thorough investigation is the long-term safety of hybrid SNAs. While numerous studies have confirmed the baseline biocompatibility of most SNA formulations, further comprehensive in vivo experiments are indispensable to rigorously evaluate their potential long-term toxicity for clinical translation. The prolonged persistence of accumulated SNAs within target cells may provoke cytotoxicity, disrupting homeostatic cellular functions and compromising cell survival. Moreover, during systemic circulation in the bloodstream, the highly charged nucleic acid shell inevitably adsorbs various serum proteins [51,52] to form a “protein corona”. This corona-bound protein layer has been verified to activate complement receptors on macrophages, thereby accelerating receptor-mediated endocytosis and triggering rapid immune clearance, which prematurely depletes the circulating SNA pool. Furthermore, due to the relatively low binding energy or weak anchoring of certain loaded DNA sequences, competitive displacement reactions are prone to occur in complex biological fluids, thereby altering the intended DNA composition. Such interconnected challenges, including protein corona formation and off-target clearance, significantly escalate the required therapeutic dosage, thereby increasing the patient’s metabolic burden. Consequently, minimizing all modalities of chronic toxicity and mitigating clinical side effects remain paramount for expanding the utility of SNAs as a mainstream, safe therapeutic modality.

#### 4.1.2. Lack of Rational Design Strategy

**Figure 7:**
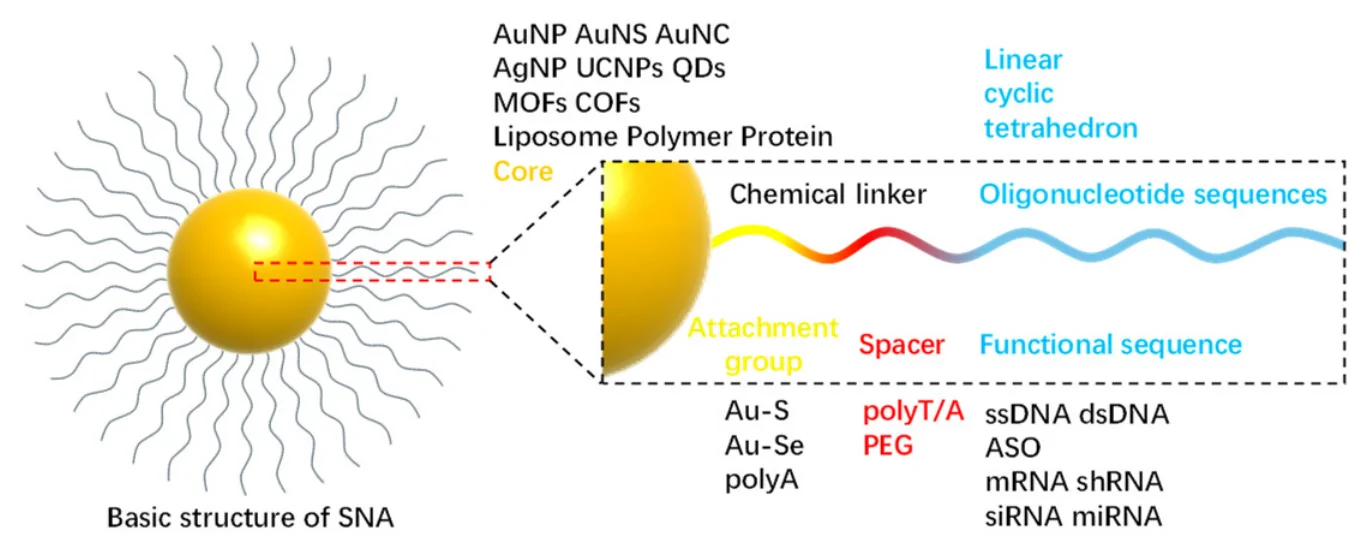
Illustration of the overall structure of SNA. For every part, several candidate instances are given to show the diversity of SNA design and construction.

When custom-designing SNAs for specific biomedical functionalities, researchers must judiciously select and optimize an array of components, including the core, oligonucleotide sequences, and chemical linkers (Figure 7). This structural tuning, coupled with labor-intensive synthesis and subsequent in vitro or in vivo evaluations, remains a highly complex and formidable process. Although prior knowledge regarding target cell properties and downstream signaling cascades can narrow down potential candidates, current development paradigms heavily rely on an iterative “synthesis-then-test” logic. Specifically, nanostructures must be physically fabricated and empirically evaluated through experiments; if the biological outcomes fall short of the predefined therapeutic goals, a completely new design cycle must be initiated. To bypass this inefficient and laborious loop, a transition toward a rational, purpose-driven design methodology is urgently required.

Recent paradigm shifts have integrated high-throughput automated synthesis with machine learning (ML) architectures to rationally guide the predictive design of SNA formulations. Yamankurt and co-workers [53] systematically altered 11 key structural attributes across multiple dimensions, encompassing core parameters (such as overall diameter and lipid composition), antigenic profiles (including antigen type, packing density, and spatial location), and oligonucleotide characteristics (encompassing sequence composition, phosphate backbone chemistry, conjugation termini, linkers, and surface density). Through this multi-parametric engineering, they established a comprehensive library comprising 960 distinct SNA formulations. This library was subsequently subjected to an automated high-throughput screening platform to quantify the resultant macrophage immune activation across four distinct concentrations with two biological replicates. These multi-dimensional datasets were then utilized to train a supervised ML model capable of mapping out the optimal design landscape for immunological applications. By leveraging this combined experimental and computational approach, the ML model successfully deciphered and predicted the structure-activity relationships of tens of thousands of hypothetical SNA permutations using a significantly reduced empirical subset (on the order of thousands).

Nonetheless, while this computational approach offers remarkable generality, it possesses inherent limitations in predictive precision. It is worth noting that this pioneering study did not directly aim to identify or optimize a final clinical immunotherapeutic candidate for a specific disease model. Crucially, critical structural determinants—such as alternative core materials or complex three-dimensional oligonucleotide folding—were omitted from the 11 baseline parameters. Furthermore, single-marker immune activation measurements alone remain an inadequate indicator to comprehensively predict the multifaceted, holistic in vivo activity of SNAs within complex biological microenvironments.

### 4.2. From Algorithm to Application: AI-Powered SNAs

**Figure 8:**
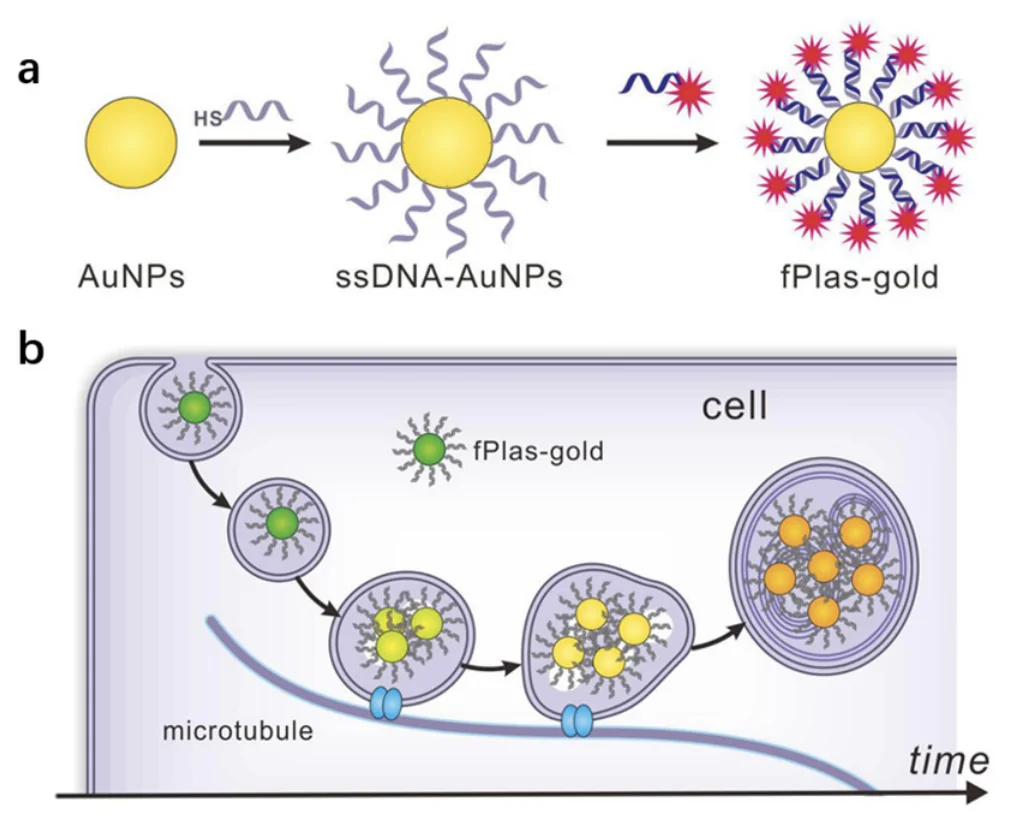
(**a**) Preparation of fPlas-gold for fluorescence microscopy and dark-field microscopy. (**b**) Schematic illustration of clustering of fPlas-gold during the intracellular traffic along microtubules. fPlas-gold nanoparticles enter cells mainly in the monomeric form and those trapped in early endosomes are gradually clustered via vesicle fusion during the maturation process. fPlas-gold nanoparticles in lysosomes exist predominantly as large clusters. Reproduced with permission [54]. Copyright 2017, Springer Nature, Licensed under CC BY 4.0.

Liu and co-workers developed an advanced computational algorithm to precisely track the endocytosis and intracellular trafficking of SNAs [54,55]. In their work, nanostructures were fabricated by passivating 50 nm AuNPs with thiolated ssDNA, followed by the hybridization of fluorophore-labeled complementary ssDNA (Figure 8a). This dually emissive design seamlessly integrated distinct fluorescent and plasmonic properties into a single bimodal probe, ensuring that the fluorescent signals remained strictly correlated with the plasmonic signatures to verify nanoparticle integrity during in situ intracellular imaging. Subsequently, the captured optical images were processed using ImageJ Fiji and decoded via a colorimetry-based algorithm executed in Python 2.7 [55]. Specifically, for these 50 nm SNAs, green, yellow, and bright-yellow/red diffraction spots corresponded to single particles, small clusters (2–5 particles), and large aggregates (>5 particles), respectively. This clustering state correlates with a monotonic redshift in the scattering wavelength: 530–570 nm for single particles, 570–580 nm for small clusters, and 580–620 nm for large aggregates. Notably, the precise aggregation states of intracellular SNAs could be efficiently differentiated within 20 s using in situ correlative dark-field and scanning electron microscopy. Concurrently, correlative dark-field and fluorescence microscopy was employed to investigate organelle co-localization and map out the holistic intracellular distribution of the nanostructures. This diagnostic platform can be readily adapted to monitor the real-time endocytosis and spatial distribution of plasmonic nanoparticles across diverse aggregation states (Figure 8b). By establishing a physical color-to-aggregation mapping, the algorithm automatically translates color profiles into specific cluster types, bypassing the need for complex spectrometers or subjective manual judgements, thereby providing a robust index for training future ML models.

Furthermore, Liu and colleagues extended the paradigm of artificial intelligence (AI)-driven therapeutic interventions [56], demonstrating that the success of SNAs in reversing liver sinusoidal endothelial cell (LSEC) capillarization was fundamentally mediated by the strategic identification of miR-325-3p from complex multi-omics datasets. To discern the therapeutically active miRNA candidates, an AI framework was established to collaboratively analyze heterogeneous omics data. Leveraging the advanced text-mining and data-structuring capabilities of ChatGPT 4.0, this computational pipeline screened human and mouse miRNA profiles along with their corresponding target downstream genes derived from Gene Set Enrichment Analysis (GSEA) and TargetScan databases. To identify key differentially expressed regulatory miRNAs, the AI model was trained on RNA-seq profiles derived from LSECs. These cells were isolated from mice with CCl_4_-induced liver fibrosis and had received either mesenchymal stem cell (MSC) or mock treatments. Concurrently, proteomics datasets derived from LSECs isolated from three healthy donors and 12 patients with clinical cirrhosis were integrated into the same computational framework. By executing these two independent analytical workflows, the AI model successfully cross-referenced the datasets to rank the top 10 miRNA candidates most likely driving these pathological variances. Remarkably, miR-325-3p emerged as the solitary consensus candidate common to both independent screening cohorts. These collective findings robustly established miR-325-3p as the pivotal therapeutic agent responsible for MSC-mediated reversal of LSEC capillarization, underscoring the power of coupling AI-assisted target discovery with functionalized SNA delivery platforms.

Following the computational cross-analysis, the pivotal regulatory role of miR-325-3p was systematically validated through empirical experiments. The authors confirmed that the reversal of MSC-mediated LSEC capillarization operates in a strictly miR-325-3p-dependent manner. This molecular mechanism was verified via in vitro transfection assays utilizing miR-325-3p mimics in LSECs, complemented by in vivo murine models involving the transplantation of MSCs modified with a miR-325 inhibitor. To achieve targeted delivery of miR-325-3p to hepatic LSECs during anti-fibrotic treatment, SNAs possessing inherent LSEC-specific internalization profiles were utilized. To maximize therapeutic efficacy, the spatial dimensions of nanostructures were systematically optimized to trigger efficient receptor-mediated endocytosis. A series of SNA formulations with hydrodynamic diameters spanning from 13 nm to 102 nm were synthesized. Subsequently, the size-dependent cellular internalization kinetics of these nanostructures into LSECs and a macrophage cell line (RAW 264.7) were evaluated via laser scanning confocal microscopy following a 6 h co-incubation period. Optical observations revealed that the 39 nm SNA formulation exhibited the highest internalization efficiency in LSECs, while demonstrating relatively low uptake by RAW 264.7 macrophages and minimal accumulation in primary Kupffer cells. Capitalizing on this exceptional delivery efficiency and cell-selective tropism, this optimized 39 nm SNA was selected for subsequent liver fibrosis therapies. Supported by this synergistic coupling of AI and experimental screening, the engineered SNA was constructed with miR-325-3p payloads on optimized 39 nm AuNPs. These SNAs effectively reversed LSEC capillarization and exhibited profound therapeutic efficacy across three distinct in vivo mouse models of liver fibrosis.

## 5. Conclusions

Significant progress has been made in the structural engineering of SNAs, primarily concerning the programmatic modulation of their dimensions, core–shell compositions, and spatial assembly regimes. Future designs could explore core-free or bio-inspired SNAs. Employing fully organic or biomimetic cores may reduce long-term tissue retention and immune interference, while preserving the programmable surface density. A particularly provocative concept is the development of programmable supramolecular SNAs that spontaneously disassemble upon completing a therapeutic cycle and can be completely metabolized by the body, thereby addressing the key safety challenge of progressive accumulation in tissues.

While these architectural milestones have laid a robust foundation for efficient delivery, they simultaneously underscore a critical transition in the field: the primary bottleneck in SNA-based therapeutics is rapidly shifting from physical vehicle optimization to the systematic discovery and functional validation of therapeutic nucleic acid sequences. This nucleic acid configurability allows SNAs to simultaneously integrate targeted delivery, sensing, and therapeutic function, enabling rational combination therapies and immunomodulation. Collectively, these advances point toward an impending paradigm shift in the nanomedicine pipeline, where high-throughput multi-omics screening, AI-driven sequence prediction, and precise pathological profiling must become standard infrastructure in nucleic acid drug development. Ultimately, advancing next-generation SNA-based interventions will increasingly depend on such systematic methodologies to prioritize which sequence to deliver over solely how to deliver it, thereby unlocking the full translational potential of polyvalent SNA platforms for targeted precision medicine.

While the identification of a therapeutically active nucleic acid payload represents a crucial starting point, it is by no means sufficient for the successful fabrication of a functional SNA. Beyond selecting a viable sequence, multiple interdependent design parameters must be simultaneously optimized to achieve effective in vivo therapy. For instance, the hydrodynamic size of the nanostructures must be systematically screened to maximize target-cell internalization while minimizing rapid macrophage clearance. Beyond simple codelivery, SNAs could be engineered to sense local therapy response and self-modulate downstream payload release, forming closed-loop therapeutic circuits. To enable a more rational engineering paradigm, big data and bioinformatics tools can be leveraged to develop predictive computational models. These frameworks can pre-calculate the spatial interactions among candidate SNA components, thereby proposing feasible assembly schemes optimized for specific therapeutic purposes.

In general, data-driven computational approaches are expected to overcome the historical lack of rational design strategies for polyvalent nanostructures. Although high-throughput techniques are long-established industry standards in the combinatorial screening of small-molecule therapeutics, such algorithmic models are only beginning to be implemented to define structure-activity relationships for functional SNAs. Predictive assays that evaluate SNA activity solely based on the isolated traits of individual nucleic acid components are inherently inaccurate. The holistic range of macromolecular interactions must be integrated into the design pipeline of SNA-based nanomedicines, particularly when applying nonlinear ML models to forecast biological responses. Unlike oversimplified linear assumptions, these advanced nonlinear architectures can successfully deconvolve the complex, cooperative effects of particle dimensions, surface chemistry, and nucleic acid topologies on cellular uptake, endosomal escape, and systemic biodistribution, ultimately enabling more precise and highly generalizable predictions.

Indeed, to realize rational approaches to SNA-based nanomedicines, the combination of high-throughput experimentation and computational analysis is necessary. With the assistance of AI, functional nucleic acid sequence selection and structure-activity relationships of SNAs can be systematically determined. All these challenges drive extensive collaboration among experts in chemistry, biology, materials science, and computer science for the development of effective SNA-based nanoarchitectures for diverse medical applications. Looking further ahead, the intrinsic programmability of SNAs could be harnessed to tailor therapeutics or diagnostics based on patient-specific microenvironment signatures or circulating biomarkers. With real-time feedback integration, one could envision personalized nucleic acid therapy that dynamically adjusts its therapeutic sequences or activation thresholds per patient. Such responsive architectures are poised to guide the next generation of SNA designs toward clinically viable, individualized nucleic acid interventions.

## Data Availability

No new data were created or analyzed in this study. Data sharing is not applicable to this article.
